# No Evidence for Dystonia-Like Sensory Overflow of Tongue Representations in Adults Who Stutter

**DOI:** 10.3389/fnhum.2019.00336

**Published:** 2019-10-04

**Authors:** Sarah M. E. Vreeswijk, T. N. Linh Hoang, Alexandra Korzeczek, Nicole E. Neef, Alexander Wolff von Gudenberg, Walter Paulus, Martin Sommer

**Affiliations:** ^1^Department of Clinical Neurophysiology, University Medical Center Göttingen, Georg-August-University, Göttingen, Germany; ^2^Department of Neuropsychology, Max Planck Institute for Human Cognitive and Brain Sciences, Leipzig, Germany; ^3^Institut der Kasseler Stottertherapie, Bad Emstal, Germany

**Keywords:** somatosensory evoked potentials, stuttering, sensorimotor integration, afferent pathway, trigeminal

## Abstract

Persistent developmental stuttering (PDS) disrupts speech fluency in about 1% of adults. Although many models of speech production assume an intact sensory feedback from the speech organs to the brain, very little is actually known about the integrity of their sensory representation in PDS. Here, we studied somatosensory evoked potentials (SEPs) in adults who stutter (AWS), with the aim of probing the integrity of sensory pathways. In addition, we tested the processing of dual sensory input to address a putative link between stuttering and focal dystonia. In 15 AWS (aged 15–55 years; three females) and 14 matched fluent speaking adults (ANS), we recorded SEPs at C5′ and C6′ induced by stimulating separately or simultaneously the tongue or the cheek at the corner of the mouth. We determined latencies (N13, P19, and N27) and peak-to-peak amplitudes (N13-P19, P19-N27). We divided amplitudes from simultaneous stimulation by the sum of those from separate stimulation. Amplitude ratios did not differ between groups, indicating normal processing of dual sensory input. This does not support a clinical analogy between focal dystonia and persistent stuttering. SEP latencies as a measure of transmission speed in sensory pathways were significantly shorter in stuttering subjects than in fluent speaking participants, however, this might have been related to a trend for a height difference between groups, and was not confirmed in a replication dataset. In summary, we did not find evidence for dystonia-like sensory overflow of tongue representations in AWS.

## Introduction

Stuttering is a speech fluency disorder characterized by involuntary repetitions or prolongations of speech sounds, and by speech blocks caused by a transient loss of speech motor control (Bloodstein and Ratner, 2008). Stuttering severity changes under stress or excitement, as in public speaking, and is reduced when the person who stutters (PWS) is in a more relaxed state or under fluency-enhancing conditions such as in chorus reading (Foundas et al., 2004). Stuttering events occur more often at the beginning of words, and are more likely to occur under high linguistic demands such as phonetic complexity, and with content words and for content-determining words (Dworzynski and Howell, 2004). The impairment is task-specific for speech, leaving other functions of orofacial muscles, such as chewing or swallowing, unaffected (Kiziltan and Akalin, 1996), even though subtle, mostly subclinical impairments of non-speech motor functions have been described (e.g., Vaughn and Webster, 1989). Movements of other body parts may accompany stuttering events (Mulligan et al., 2001).

Persistent developmental stuttering (PDS) concerns all the above-mentioned symptoms occurring from an early age and persisting into adulthood. Men are affected four times more often than women (Bloodstein and Ratner, 2008). It is among the most frequent speech fluency disorders, affecting about 1% of the adult population (Yairi and Ambrose, 1999). Many hypotheses have been proposed as to the origin of PDS (Büchel and Sommer, 2004). A basal ganglia involvement is suggested by positive treatment effect of antidopaminergic drugs (Brady, 1991).

We here explore the view that stuttering shares features with focal dystonias (Kiziltan and Akalin, 1996). A focal dystonia is a task-specific disorder of fine sensorimotor control. Performing a motor task, such as writing in so-called writer’s cramp, induces an excessive activation of task-related and task-unrelated muscles, resulting in dysfunctional posturing or twitches impairing task execution. It is accentuated by emotional stress (Hallett, 1995; Berardelli et al., 1998; Morgante et al., 2011). While the motor cortical characteristics of PDS appear to differ from those of focal dystonias (Neef et al., 2011), little is known regarding the sensory domain. Focal dystonias, such as writer’s cramp or musician’s cramp, may be associated with an altered representation of the affected limb on the somatosensory cortex (Nelson et al., 2009). Stuttering and dystonia share several neural features (Ludlow and Loucks, 2003; Alm, 2004). Especially sensory effects show some parallels. An attenuation of sensory feedback, such as altered auditory feedback in stuttering and tactile sensory stimulation of the part of the body affected from dystonia, reduce symptoms (Alm, 2004). Thus, we speculated that inhibitory integration of afferent inputs may be deficient in stuttering, as has been shown for focal dystonias.

One way to assess the handling of sensory input is by measuring Somatosensory Evoked Potentials (SEP). This is a routine clinical procedure to assess the integrity of somatosensory pathways (Stoehr, 1996). It has been elaborated into a neurophysiological test of cortical inhibition in dystonias by using a dual stimulation method (Tinazzi et al., 2000). By simultaneously stimulating two nerves of the hand, the median and ulnar nerve, in patients with dystonia involving at least one upper limb, they found an abnormal integration of sensory input. The relative SEP amplitude increase in dual as compared to single stimulation was much larger in the patient group than in the control group. The interpretation was that the inhibitory capacity of the sensory system to control and to limit the relative sensory overflow caused by simultaneous stimuli was impaired in these dystonia patients (Tinazzi et al., 2000). By contrast, SEP latencies were unchanged in their study. Tinazzi et al. (2000) related this deficient inhibitory control of afferent input to the motor impairment in dystonia. Thus, a deficient inhibitory integration of afferent inputs as shown in dystonia (Tinazzi et al., 2000) might cause a signal overflow in sensorimotor loops, and reducing the strength of feedback might reduce the risk for signal overflow (Alm, 2004).

We used this methodology to answer our hypothesis by combining SEP of the cheek with SEP of the tongue, thus attempting to quantify speech muscle related sensory cortex activation patterns. We hypothesized that our research would show a similar decreased capacity of integrating the dual sensory input in PWS when compared with persons who do not stutter (PNS) if there was common ground between focal dystonia and stuttering. Also, we expected SEP latencies to be normal in PWS since latency deviations had only been reported for event-related potentials (Beal et al., 2010).

## Materials and Methods

The protocol was approved by the University Medical Center Göttingen ethics committee, and we obtained written informed consent before any study-related procedure took place.

### Participants

We investigated 15 subjects with PDS whose clinical characteristics are shown in Table 1. They were recruited from the “Institut der Kasseler Stottertherapie” (Euler et al., 2009) and the Göttingen stuttering support group. Their data were compared with those from 15 matched healthy PNS with no personal or family history of stuttering. In one control subject, no reproducible SEP recordings could be elicited by tongue stimulation, and this subject was therefore not included in data analysis. None of the participants had any unstable medical or neurological prior illnesses, and none of them were taking CNS-active drugs at the time of participation. In all participants, we determined age and body height since they are known to influence SEP latencies (Chiappa, 1990; Stoehr, 1996). Based on two video samples of spontaneous speech as well as reading, the participants’ speech fluency was assessed by a qualified speech-language pathologist using the German version of the stuttering severity instrument (SSI-3; Sandrieser and Schneider, 2008).

**Table 1:** Characteristics of participants.

Measures	Stuttering	Control	Significance
Participants, *n*	15 (12 M, 3 F)	14 (11 M, 3 F)	-
Age in years, mean	28.07 (SD = 12.17)	30.50 (SD = 7.80)	*p* = 0.52 (n.s.)
Handedness, mean	69.84 (SD = 60.13)	75.26 (SD = 38.54)	*p* = 0.74 (n.s.)
Body height, cm	177.40 (SD = 8.84)	183.79 (SD = 10.57)	*p* = 0.09 (n.s.)
Percentage of syllables stuttered, mean	9.55 (SD = 6.06)	0.61 (SD = 0.82)	*p* < 0.001 (sig.)

*Comparison of age, gender, body height, handedness and percent of stuttered syllables of all participants. Groups were compared using unpaired two-tailed t-test, and with Mann-Whitney U-test for handedness and percentage of syllables stuttered*.

### SEP Recordings

Right and left facial and tongue SEPs were recorded while the participants sat in a reclining chair. The cheek was stimulated at the corner of the mouth (maxillary and mandibulary branch) with a stimulating electrode composed of cotton bars (Digital Stim Electrode 5032-TP, Viasys Inc., Madison, WI, USA) soaked in saline solution for improved conduction, with electrical square pulses of 0.2 ms duration at a rate of 5.1 Hz, i.e., at an interpulse interval of 196 ms (Kimura, 1989b). Stimuli were delivered at motor threshold intensity, inducing a barely noticeable twitching of the upper lip. The tongue was stimulated with gold cup electrodes attached to a mouthpiece and falling into place on the right and left upper lateral side of the tongue, with one touching down near the tip of the tongue and one 25 mm further to the back on either side Again, electrical square pulses of 0.2 ms duration at a rate of 5.1 Hz were used, at an intensity slightly noticeable by the participants yet below a painful level. We used a spoon-like mouthpiece adapted from earlier studies (Rodel et al., 2003; Neef et al., 2011). The dimensions of the mouthpiece allowed the attached electrodes to fall upon the upper surface of tongue without additional muscle tension of the jaw or tongue required to keep it in place. In random order, we tested three modes of stimulation on either side: the cheek stimulated alone (Chk), the tongue stimulated alone (To), and both sites stimulated simultaneously (ToChk). Each mode was tested in two consecutive runs of 500 pulses each, with reversal of polarity after half of the 500 pulses of each run to minimize baseline shifts due to excessive stimulus artifacts. SEPs were recorded in a resting state. Participants were not given a task; they were asked to lay calm and relaxed. Audio feedback from the EMG channels was provided to monitor a relaxed muscle state.

Recording electrodes were placed according to the international 10–10 system over C5′ and C6′, corresponding to the orofacial area of the left and right somatosensory cortex, respectively, which were referenced to Fz. SEPs were recorded using a Nicolet VikingSelect with software version 11.1 and a Nicolet ET 16 headbox, amplified using a Nicolet ES-8 amplifier and filtered at 2 Hz and 1 kHz (all equipment Viasys healthcare systems, now CareFusion Inc., Waukegan, IL, USA). We did not activate a notch filter.

### SEP Analysis

Though nowadays used less often in clinical practice, the trigeminal nerve SEP serves as an investigative tool in clinical studies, and the recorded potential shows a triphasic pattern of negative-positive-negative named N13, P19, and N27. It is cortical in origin (Bennett et al., 1987) and, in analogy to hand nerve stimulation-induced SEPs, thought to be generated in the primary sensory cortex (Allison et al., 1991; Buchner et al., 1996). Reports of successful SEP recordings from tongue stimulation (Altenmüller et al., 1990) gave us the inspiration to combine SEP recordings from two stimulation sites of the orofacial region, namely the tongue and the cheek near the upper lip, in an adaptation of Tinazzi’s method in an attempt to quantify speech muscle related sensory cortex activation patterns.

For analysis, the peak latencies N13, P19, and N27 of the cortical potential elicited by stimulation were determined automatically and corrected manually in case of obvious misplacement, and the peak-to-peak amplitudes of N13-P19 and P19-N27 were calculated automatically by the VikingSelect software. The peak-to-peak ratio was calculated as:

$$\text{P}-\text{P}-\text{amp}\frac{\text{ToChk}}{\text{To+Chk}}$$

where ToChk is the SEP amplitude obtained after simultaneous stimulation of the tongue and the cheek, and To + Chk is the sum of the SEP amplitudes obtained after individual stimulations of the aforementioned sites (Tinazzi et al., 2000). Contralateral recordings were used for analysis.

### Statistical Analysis

Groups were compared using unpaired, two-tailed *t*-tests for age, stimulation intensity, and body height, and with Mann-Whitney *U*-tests for handedness and for percentage of syllables stuttered.

Raw amplitudes were analyzed in a mixed-design ANOVA with “group” as between-subjects-factor, and “side of stimulation” (left, right), “run” (run 1, run 2), “type of stimulation” (tongue alone, cheek alone, simultaneous), and “amplitude” (N13-P19, P19-N27) as within-subjects factors. Amplitude ratios were analyzed using a mixed-design ANOVA, with “group” as between-subjects-factor, and “side of stimulation” (left, right), “run” (run 1, run 2), and “ratio” (N13-P19, P19-N27) as within-subjects factors. Raw latencies were analyzed in a mixed-design ANOVA, with “group” as between-subjects-factor, and “side of stimulation” (left, right), “run” (run 1, run 2), “type of stimulation” (tongue alone, cheek alone, simultaneous), and “latency” (N13, P19, N27) as within-subjects factors. We analyzed the stimulus intensities using a mixed-design ANOVA, with “group” as between-subjects-factor, and “side of stimulation” (left, right), “site of stimulation” (tongue, cheek) as within-subjects factors.

In addition, we correlated the pooled SEP latency with age body height, percentage of syllables stuttered, and with the pooled stimulus intensities using the STATVIEW 5.0 regression function and *F*-tests.

For all analyses, age was calculated in days, subtracting the day of birth from the day of measurement (Microsoft Excel). SPSS 20 was used for all other statistics. In all ANOVAs, *post hoc*, unpaired, two-tailed *t*-tests were done based on significant main effects. *P*-values of < 0.05 were considered significant.

### SEP Latency Replication Study

To corroborate the unexpected findings with regards to SEP latencies, we performed a replication study on eight ANS (two females; average age 26.25 years SD 4.03; average height 179.25 cm SD 8.78) and seven adults who stutter (AWS; no females, average age 24.86 years SD 4.60; average height 181.29 cm SD 8.32; unpaired, two-tailed *t*-test for age, *p* = 0.55; for height, *p* = 0.65), none of whom had been part of the principal experiment. Again, we analyzed raw latencies in a mixed-design ANOVA with “group” as the between-subjects-factor, and “side of stimulation” (left, right), “run” (run 1, run 2), “type of stimulation” (tongue alone, cheek alone, simultaneous), and “latency” (N13, P19, N27) as within-subjects factors.

## Results

**Figure 1:**
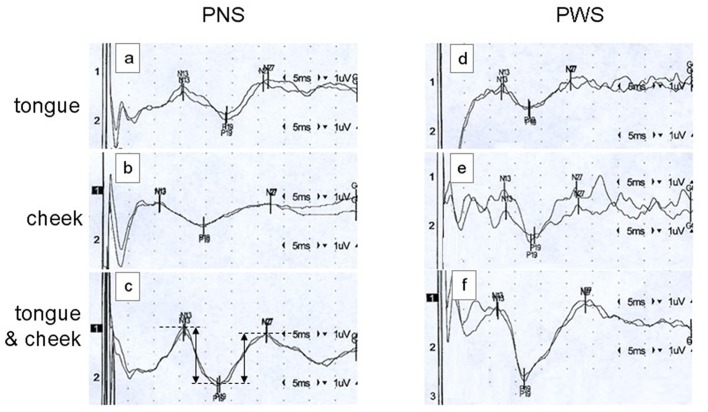
Examples of somatosensory evoked potentials (SEPs) elicited by tongue alone stimulation (traces **A,D**), cheek alone stimulation (traces **B,E**), or simultaneous tongue and cheek stimulation (traces **C,F**) in a 26-year old fluid speaker (PNS; traces **A–C**) and a 26-year old person who stutters (PWS; traces **D–F**). Each trace constitutes the average of 500 pulses, with reversal of polarity after 250 ms to minimize the stimulus artifact. Stimulation on the right side and recording over the contralateral cortex at C5′. In trace **(C)**, the dimensions of the peak-to-peak amplitudes N13-P19 and P19-N27 as calculated automatically are illustrated.

A typical example of SEP recordings is shown in Figure 1. It shows tongue, cheek, and simultaneous tongue and cheek stimulation in a PNS and in a PWS. Note the significant and variable stimulus artifacts which do not impair the detection of the peak latencies. Latencies are shorter in the PWS than in the PNS, particularly with tongue alone and with simultaneous stimulation.

**Figure 2:**
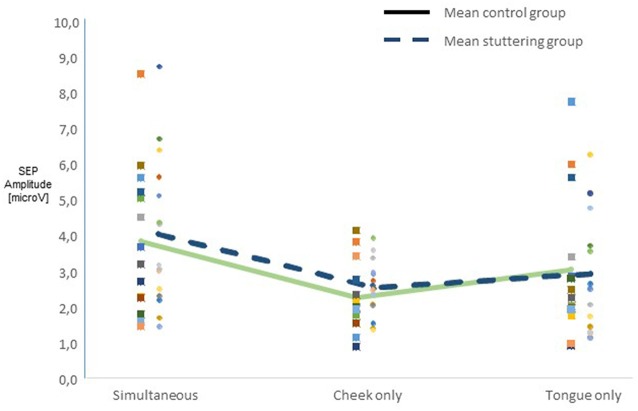
Integration of dual sensory input. SEP amplitudes N13-P19 and P19-N27 in 15 adults who stutter (AWS; hatched lines) and 14 adults who do not stutter (solid lines). Pooled amplitudes for each type of stimulation as noted on the abscissa. The simultaneous stimulation yielded larger SEP amplitudes than the other types of stimulation.

Across all participants, raw amplitudes yielded a main effect of type of stimulation (*F*_(2,54)_ = 10.45, *p* < 0.0001). *Post hoc* *t*-tests confirmed higher amplitudes in the simultaneous condition than in any of the other types of stimulation (Figure 2). There was no main effect of group, though, and no two-factor interaction of group with any other factor. Across groups, side of stimulation interacted with amplitude (*F*_(2,54)_ = 42.58, *p* < 0.0001, Supplementary Figure S1A), *post hoc* tests confirmed larger N13-P19 than P19-N27 amplitudes after right-sided stimulation and vice versa after left-sided stimulation. Type of stimulation and amplitude interacted significantly (*F*_(2,54)_ = 5.51, *p* = 0.007, Supplementary Figure S1B), *post hoc* tests confirmed larger N13-P19 than P19-N27 amplitudes with cheek stimulation.

**Figure 3:**
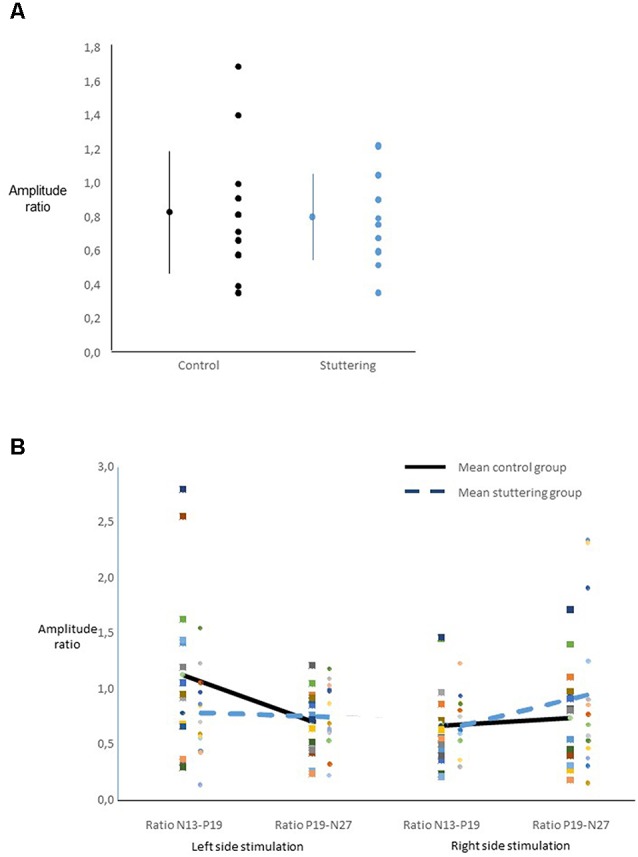
Ratio of SEP amplitudes. For calculation see text. **(A)** For each group, the dot and bars on the left show the mean ± one standard deviation, the multiple dots on the right show the individual values. There was no main effect of group. **(B)** Interaction of ratio by side of stimulation across groups.

Ratios of amplitudes did not yield a main effect of group (Figure 3A), nor any other significant main effect. Across groups, there was an interaction of side of stimulation by ratio (*F*_(1,27)_ = 6.40, *p* = 0.018, Figure 3B), with *post hoc* *t*-tests showing a larger N13-P19 ratio than P19-N27 ratio after right-sided stimulation, and vice versa after left-sided stimulation.

Raw latencies yielded a main effect of group (*F*_(1,27)_ = 4.39, *p* = 0.046), with *t*-tests confirming shorter latencies PWS than in PNS. Also, there was a main effect of type of stimulation (*F*_(2,54)_ = 4.78, *p* = 0.012), with *post hoc* tests indicating shorter latencies in the cheek alone than in the tongue alone condition. We found an interaction of type of stimulation by group (*F*_(2,54)_ = 4.12, *p* = 0.022, Supplementary Figures S2A,B). *Post hoc* *t*-tests showed no differences between types of stimulation among PWS, whereas among PNS, simultaneous stimulation, tongue stimulation, and cheek stimulation differed from each other. There was also an interaction of side of stimulation by latency (*F*_(2,54)_ = 11.66, *p* < 0.0001), with the P19 being slightly longer after left than after right-sided stimulation, and vice versa for N13 and N27. Of course, there was a main effect of latency (*F*_(2,54)_ = 2,274.6, *p* < 0.0001). The interaction of type of stimulation by group was confirmed in an ANCOVA, taking into account body height and age as covariates (see Appendix 1).

Tc between groups (effect of group, *F*_(1,27)_ = 5.05, *p* = 0.033), being lower in PWS than in PNS (Supplementary Figure S3). There were no other main effects or interactions.

Across groups, pooled SEP latencies increased with age (*r* = 0.085, *F*_(1,2086)_ = 15.30, *p* < 0.0001) and with body height (*r* = 0.111, *F*_(1,2086)_ = 25.93, *p* < 0.0001). SEP latency did not change with tongue stimulation intensity, but latency did increase with increasing left (*r* = 0.059, *F*_(1,2086)_ = 7.64, *p* < 0.0067) and right (*r* = 0.077, *F*_(1,2086)_ = 12.34, *p* = 0.0005) cheek stimulation intensity. The percentage of syllables stuttered correlated with neither SEP latency nor stimulus intensity.

In the independent replication sample, raw SEP latencies were similar between groups [effect of group (*F*_(1,13)_ = 0.84, *p* = 0.38)]. There was a main effect of type of stimulation (*F*_(2,26)_ = 6.38, *p* < 0.006). Except for an obvious effect of SEP (*F*_(2,26)_ = 936.40, *p* < 0.0001), no other effects or interactions were significant. We also performed a median/ulnar nerve control study in a subgroup of participants (Appendix 3).

## Discussion

We studied SEP amplitudes and latencies with dual stimulation in PNS and PWS. We found normal amplitude ratios to dual stimulation, refuting our hypothesis of sensory overflow caused by simultaneous dual stimulation. Hence, this finding does not support the clinical analogy of PDS and dystonia. Unexpectedly, all trigeminal SEP components were shortened, and the stimulation threshold reduced in PWS as compared to PNS, but this was not confirmed in a replication study.

### SEP Amplitudes and Latencies

So far, a role of sensory structures in individuals afflicted with PDS is controversial. While auditory feedback has a profound impact on speech fluency (Antipova et al., 2008), inconclusive findings are reported for oral stereognosis (Jensen et al., 1975; Martin et al., 1981) and vibrotactile magnitude production (Fucci et al., 1985). An impairment of kinesthetic control in PWS was suggested by larger minimal displacements of the jaw, tongue, and lips in the absence of visual feedback in PWS; and it was remedied by providing visual feedback (De Nil and Abbs, 1991). By contrast, PWS showed an even better resistance to simultaneous disturbances in the auditory, proprioceptive, and tactile domain than PNS (Namasivayam et al., 2009).

Many models of fluent speech production (Levelt et al., 1999; Guenther et al., 2006) assume a sensory feedback of the current state of the vocal tract and the articulatory muscles (e.g., Figure 4 in Hickok, 2012), attributing speech dysfluencies to mismatches in feedback or feedforward loops (Tourville and Guenther, 2011). Hence, we were initially intrigued by the unexpected latency difference, which would also have fit into the literature of white matter changes in the corticospinal tract (Cai et al., 2014; Connally et al., 2014; Kronfeld-Duenias et al., 2016). However, we might have been mistaken by a height effect, as the initial sample showed an almost significantly shorter stature of AWS than of ANS (see Table 1). Since SEP latencies increase with body height (Maurer and Eckert, 1999), this almost significant group difference might explain the latency difference. Indeed, the ANCOVA in Appendix 1 implies that the main effect of group is much weaker if height is taken into account as a covariate. In addition, even though its study population was small, and given that replication studies have numerous limitations (Anderson and Maxwell, 2017), our replication study nevertheless confirmed that the latencies were similar in AWS and ANS.

We are not aware of a study on orofacial SEP latencies in AWS. Strikingly, auditory evoked potential (AEP) latencies are atypical in AWS. Peak latencies of early auditory components have an increased variability, are prolonged, and tend to interaural differences in persons who stutter as compared to persons who do not stutter (Tahaei et al., 2014; Gonçalves et al., 2015). Beal et al. (2010) found longer latencies overall in AWS compared to ANS in a vowel listening task. Only in a sub-task of active vowel production, right hemispheric ERP latency was shorter in AWS than in ANS. The authors interpret their findings of a right hemispheric latency advantage in active vs. passive tasks as consistent with a stronger right hemispheric involvement in stuttering (Travis, 1978). Another ERP study on speech preparation did not report latency differences in AWS as compared to ANS (Daliri and Max, 2015) for late cortical components.

### Processing of Dual Input

The main finding in this study is a negative one: since our results show similar amplitude ratios in both groups, we conclude that cortical processing of dual sensory input is normal in PWS. Hence, our results do not yield positive evidence to support the hypothesis that developmental stuttering is a form of dystonia. This is consistent with recent data on intracortical inhibition as assessed by paired-pulse transcranial magnetic stimulation. It is known to be markedly reduced in focal dystonias and many other movement disorders (Ziemann and Hallett, 2000), but it was only marginally affected in AWS (Neef et al., 2011). By contrast, intracortical facilitation, which is known to be unchanged in focal dystonias and other movement disorders (Ziemann and Hallett, 2000), turned out to be strikingly reduced in AWS, thereby showing a pattern of neurophysiological abnormalities in PDS quite distinct from focal dystonias (Neef et al., 2011).

Alm (2004) discusses parallels between dystonia and stuttering in detail. One parallel is an assumed excessive sensory feedback gain, putatively remedied by removing or reducing sensory feedback. As we did not find evidence for sensory overflow in dual stimulus processing, we think that we can exclude altered sensory feedback gains as a major player in the pathogenesis of stuttering.

Clinically, there is also a subtle difference regarding the role of sensory input in the two disorders. A “geste antagoniste,” also known as a sensory trick, is a characteristic feature of many patients with focal dystonia, i.e., a light touch on a particular body part, often the cheek, moderating the excessive muscle hyperactivity, thereby transiently alleviating symptoms. This phenomenon of alleviating ongoing symptoms does not usually exist in PDS. Here, external auditory rhythm or touch often help to overcome start hesitations prior to the emergence of stuttering symptoms (Alm, 2004).

## Limitations

We did not assess a group of patients with embouchure dystonia, which would have been an appropriate additional group of study. Also, we did record SEP at rest, without active speaking condition. Hence, our conclusions are limited to trait rather than state markers of stuttering (Vanhoutte et al., 2016).

All PWS studied here had undergone speech therapy at some point, where they had been instructed to pay careful attention to the manner in which they give shape to sounds, using that to overcome dysfluencies. We cannot rule out that such increased awareness of orofacial structures might have modulated cortical representations (Pascual-Leone and Torres, 1993) and might have influenced SEP amplitude or latency.

The detection of SEP peaks can be somewhat difficult, in particular when stimulating facial areas (Stoehr, 1996). However, the baseline shift assessment did not yield any effect of group either (Appendix 2), supporting the SEP detection of the initial analysis. In addition, determining the stimulation intensity according to subjective perception might introduce a bias in group comparisons.

Unfortunately, data on the origin of the different components and amplitudes of the trigeminal SEPs are scarce, which makes it difficult to draw conclusions from the differential modulation of amplitudes we observed.

Since sensory representations of tongue and lips are adjacent and strongly overlapping (McCarthy et al., 1993), an artifact of suboptimal positioning of the recording electrodes is unlikely.

## Conclusion

We were able to overcome the technical challenges of tongue and cheek SEP recordings, and we provided detailed tools for analysis. However, the hypothesis motivating our endeavor was not met: We did not find evidence for dystonia-like sensory overflow of tongue representations in AWS. Thus enhanced sensory feedback gain of the tongue as a cause for stuttering (Alm, 2004) is not supported.

## Data Availability Statement

Pseudonymized data can be accessed by future researchers upon reasonable request based on standard hospital practices.

## Ethics Statement

This study was carried out in accordance with the recommendations of the Ethics committee of the University Medical Center Göttingen with written informed consent from all subjects. All subjects gave written informed consent in accordance with the Declaration of Helsinki. The protocol was approved by the Ethics committee of the University Medical Center Göttingen.

## Author Contributions

SV recorded the data, initiated data analysis, and wrote the first draft. TH was active in participant recruitment and data recording. AK recruited participants for the replication study and conducted the experiments. NN contributed to study design, analyzed the speech samples, and discussed the results. AWG helped with patient recruitment and refined the setup. WP provided funding and commented on the analysis and interpretation of the data. MS designed the experiments, built the setup, contributed to the statistical analysis of the data, and helped writing the manuscript. All authors discussed the results and commented on the manuscript.

## Conflict of Interest

The authors declare that the research was conducted in the absence of any commercial or financial relationships that could be construed as a potential conflict of interest.

## Supplementary Material

FIGURE S1 Integration of dual sensory input. SEP amplitudes N13-P19 and P19-N27 in 15 adults who stutter and 14 adults who do not stutter. Boxes show the median and are limited by 25th and 75th percentile, horizontal bars indicate 10th and 90th percentile, and filled circles indicate outliers. Asterisks indicate differences on *post hoc* *t*-tests. **(A)** Interaction of amplitude by side of stimulation. **(B)** Interaction of amplitude by type of stimulation.

FIGURE S2 SEP latencies. Box plots as in Supplementary Figure S1. **(A)** Main effect of group. **(B)** Interaction of group with type of stimulation. The cheek alone stimulation yielded similar SEP latencies in both groups. However, with tongue alone or simultaneous tongue and cheek stimulation, SEP latencies were shorter in adults who stutter than in the control group. Asterisks indicate significant difference between groups, circles indicate significant differences within the control group.

FIGURE S3 Stimulation intensities. Box plots as in Supplementary Figure S1. Stimulation intensities used in for SEP recording, values from cheek and tongue stimulation did not differ significantly and were pooled. For details on stimulation methods see text. Note lower stimulation intensities in the group of adults who stutter. Asterisk indicates a significant difference between groups.

FIGURE S4 Baseline shift approximation. Box plots as in Supplementary Figure S1. Approximation of baseline shift (an inherent technical problem of trigeminal SEP recording). Shift was larger in the simultaneous condition than in the separate stimulation conditions, and not different between groups. For details see text.

FIGURE S5 SEP latency from median/ulnar nerve control study. Box plots as in Supplementary Figure S1. SEP latency (mean of N20, P25 and N35) for each type of stimulation as noted on the abscissa, in five adults who stutter and five adults who do not stutter. For the hand sensory representation, latencies were not shorter in adults who stutter than in those who do not stutter.

## Appendix 1

### ANCOVA with Age and Body Height as Covariates

To further take into account eventual interfering effects of age or body height, we calculated an additional repeated-measures ANCOVA for the raw SEP latencies. As explained above, we used “group” as the between-subjects-factor, and “side of stimulation” (left, right), “run” (run 1, run 2), “type of stimulation” (tongue alone, cheek alone, simultaneous), and “latency” (N13, P19, N27) as within-subjects factors. We here added the covariates “age” (in a precision of the number of days between the day of birth and day of the recording of the current study in a given participant) and “body height” (in centimeters), using SPSS general linear model with repeated measurements and covariates (SPSS 20, IBM Inc.).

This additional analysis missed a main effect of group (*F*_(1,25)_ = 1.82, *p* = 0.189), but confirmed an interaction of type of stimulation by group (*F*_(2,50)_ = 3.83, *p* = 0.028), and a main effect of latency (*F*_(2,50)_ = 5.72, *p* < 0.006), with no other main effect or interaction. Only taking age as a covariate almost yielded a main effect of group (*F*_(1,26)_ = 3.79, *p* = 0.063), whereas only taking height as a covariate largely missed the main effect of group (*F*_(1,26)_ = 2.24, *p* = 0.147).

## Appendix 2

### Baseline Shift Approximation

Due to the proximity of stimulation and recording sites, SEP recordings from cheek or tongue are often afflicted with large stimulus artifacts resulting in considerable baseline shifts (Kimura, 1989a). While peak latencies can be expected to be quite robust to this inherent technical problem (Stoehr, 1996), these baseline shifts may blur peak-to-peak amplitude measurements. In order to approximate this shift, an additional marker was placed in each trace at 49 ms, named arbitrarily G49, and the angle or slope between this G49 and N13 was calculated as follows. In a right-angled triangle the baseline slope was translated as the inverse tangent of the ratio of the opposing leg and the adjacent leg. The opposing leg was determined automatically as the N13G49 peak-to-peak amplitude. The adjacent leg was calculated by subtracting the N13 latency from 49 ms. The estimation of the slope being upwards or downwards was determined manually, resulting in a positive factor for an upwards slope and a negative factor for a downwards slope. For all recordings the baseline slope was calculated by the following formula, tan-1[N13G49/(49-N13latency)] multiplied by 1 or −1 for either an upwards or downwards slope, respectively. Baseline slopes were not used to correct amplitudes, but were taken as such and compared between groups using a mixed-design ANOVA with “group” as the between-subjects-factor, and “side of stimulation” (left, right), “run” (run 1, run 2), and “type of stimulation” (tongue alone, cheek alone, simultaneous) as within-subjects factors.

For the baseline drift, we did not find a main effect of group, or any interaction involving group. The baseline drift was stronger with right-sided than with left-sided stimulation (effect of side of stimulation, *F*_(1,28)_ = 10.32, *p* = 0.0033). In addition, it depended on the type of stimulation, being strongest with simultaneous stimulation (effect of type of stimulation, *F*_(2,56)_ = 5.56, *p* = 0.006, Supplementary Figure S4).

## Appendix 3

### Median and Ulnar Nerve Control Study

To test the specificity of the latency difference for articulatory muscles, we rescheduled five subjects from each group of the primary experiment to repeat the experiment, stimulating the median and ulnar nerve separately or simultaneously, as originally described by Tinazzi et al. (2000). Separate runs were used for either hand. The set-up, stimulation and recording procedures were identical to those of the principal experiment; except for the use of the recording sites C3′ and C4′.

We used a mixed-design ANOVA with “group” as between-subjects-factor, and “side of stimulation” (left, right), “run” (run 1, run 2), and “type of stimulation” (median nerve alone, ulnar nerve alone, simultaneous) as within-subjects factors. SEP latencies revealed no effect of group and no two-factor interaction of group with any other factor (Supplementary Figure S5).
