# Supplementary figures and images for: No Evidence for Dystonia-Like Sensory Overflow of Tongue Representations in Adults Who Stutter
Source: Front Hum Neurosci. 2019 Oct 4;13:336. doi: 10.3389/fnhum.2019.00336 (PMC6787140; doi:10.3389/fnhum.2019.00336)

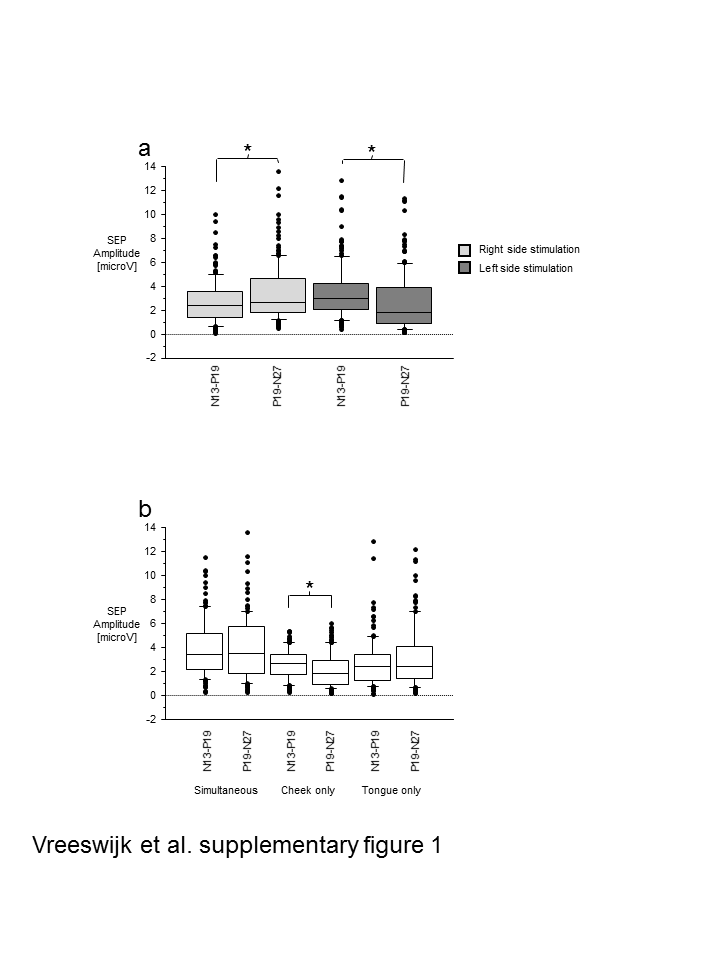

Supplement: FIGURE S1 — Integration of dual sensory input. SEP amplitudes N13-P19 and P19-N27 in 15 adults who stutter and 14 adults who do not stutter. Boxes show the median and are limited by 25th and 75th percentile, horizontal bars indicate 10th and 90th percentile, and filled circles indicate outliers. Asterisks indicate differences on post hoc t-tests. (A) Interaction of amplitude by side of stimulation. (B) Interaction of amplitude by type of stimulation. [file Image_1.TIF]

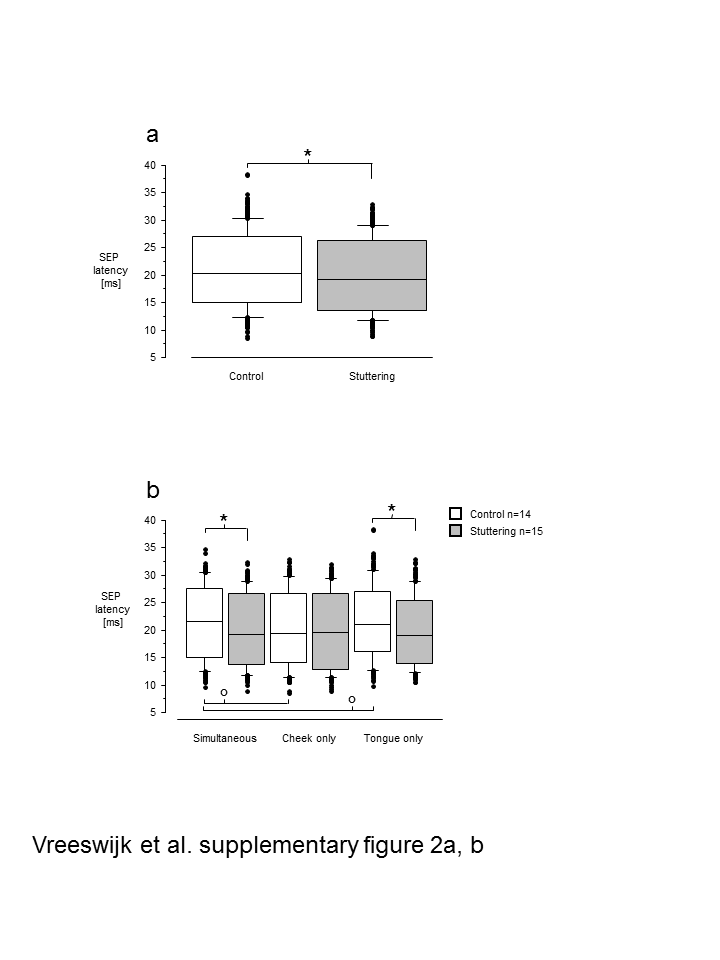

Supplement: FIGURE S2 — SEP latencies. Box plots as in Supplementary Figure S1. (A) Main effect of group. (B) Interaction of group with type of stimulation. The cheek alone stimulation yielded similar SEP latencies in both groups. However, with tongue alone or simultaneous tongue and cheek stimulation, SEP latencies were shorter in adults who stutter than in the control group. Asterisks indicate significant difference between groups, circles indicate significant differences within the control group. [file Image_2.TIF]

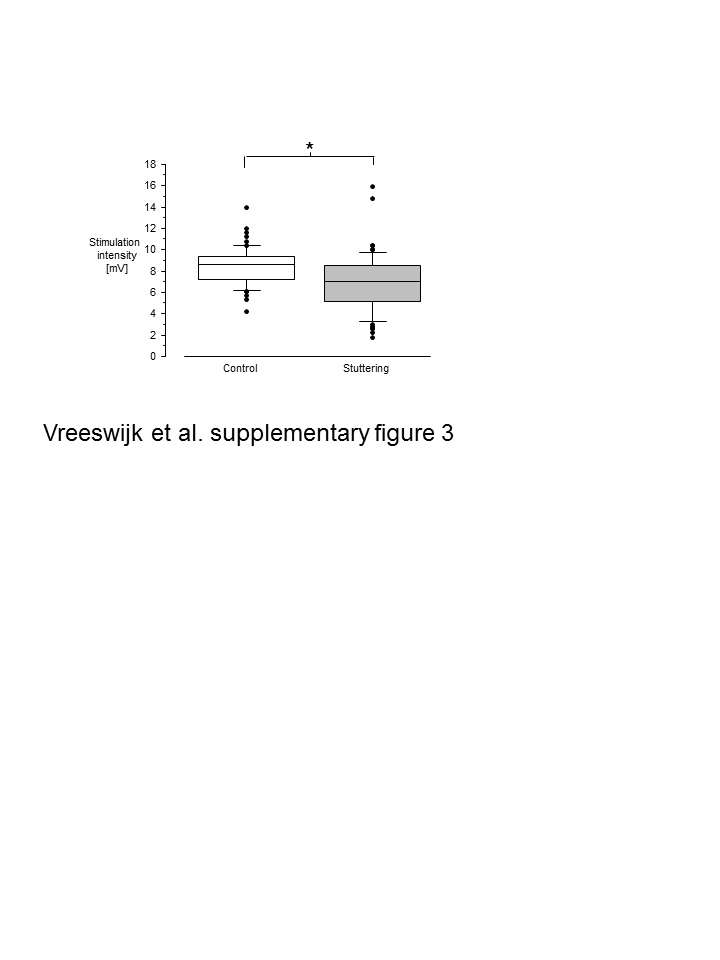

Supplement: FIGURE S3 — Stimulation intensities. Box plots as in Supplementary Figure S1. Stimulation intensities used in for SEP recording, values from cheek and tongue stimulation did not differ significantly and were pooled. For details on stimulation methods see text. Note lower stimulation intensities in the group of adults who stutter. Asterisk indicates a significant difference between groups. [file Image_3.TIF]

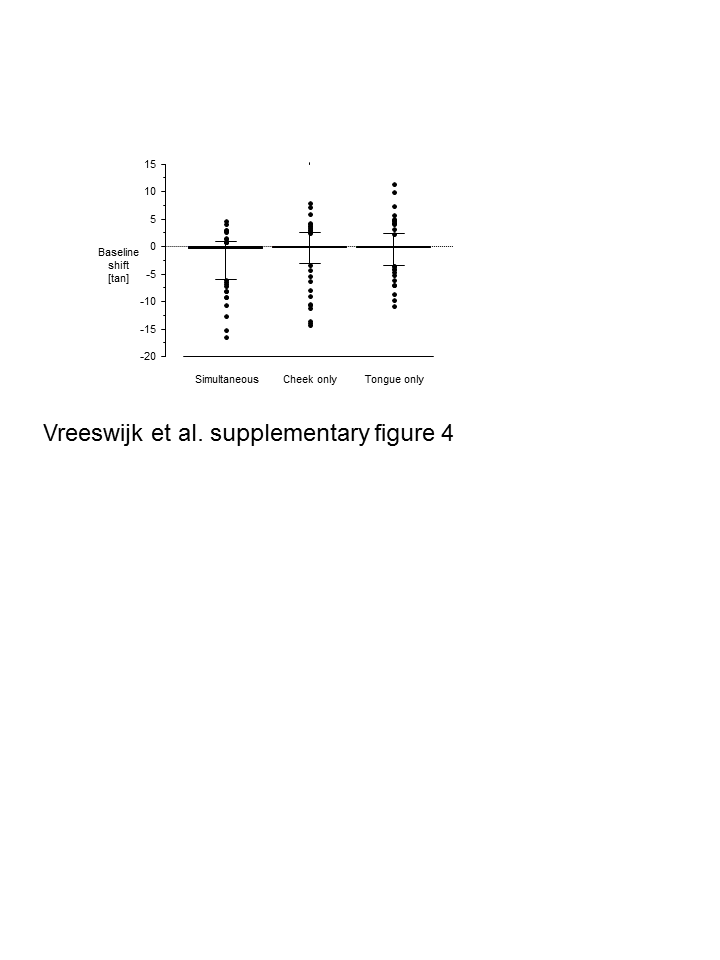

Supplement: FIGURE S4 — Baseline shift approximation. Box plots as in Supplementary Figure S1. Approximation of baseline shift (an inherent technical problem of trigeminal SEP recording). Shift was larger in the simultaneous condition than in the separate stimulation conditions, and not different between groups. For details see text. [file Image_4.TIF]

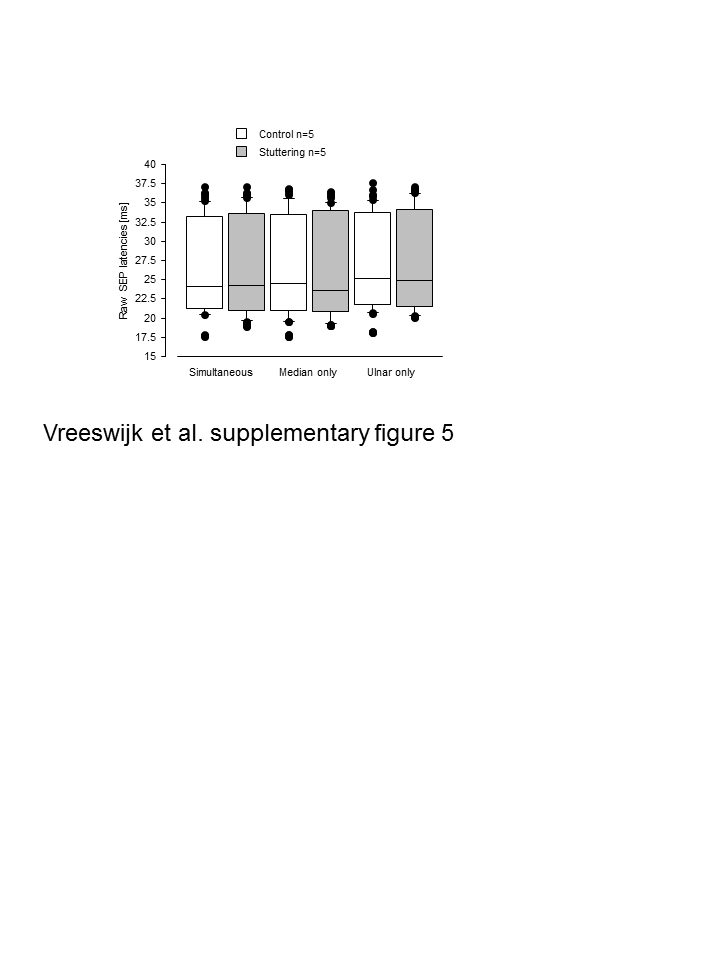

Supplement: FIGURE S5 — SEP latency from median/ulnar nerve control study. Box plots as in Supplementary Figure S1. SEP latency (mean of N20, P25 and N35) for each type of stimulation as noted on the abscissa, in five adults who stutter and five adults who do not stutter. For the hand sensory representation, latencies were not shorter in adults who stutter than in those who do not stutter. [file Image_5.TIF]
